# Polo-like kinase 1 (PLK1) inhibition suppresses cell growth and enhances radiation sensitivity in medulloblastoma cells

**DOI:** 10.1186/1471-2407-12-80

**Published:** 2012-03-05

**Authors:** Peter S Harris, Sujatha Venkataraman, Irina Alimova, Diane K Birks, Andrew M Donson, Jeffrey Knipstein, Adrian Dubuc, Michael D Taylor, Michael H Handler, Nicholas K Foreman, Rajeev Vibhakar

**Affiliations:** 1 Department of Pediatrics and Children's Hospital Colorado, University of Colorado Denver, Anschutz Medical Campus, Aurora, CO, USA; 2 Division of Pediatric Neurosurgery, Children's Hospital Colorado, University of Colorado Denver, Anschutz Medical Campus, Aurora, CO, USA; 3 Division of Neurosurgery, Program in Developmental and Stem Cell Biology, Hospital for Sick Children, Toronto, ON, Canada; 4 Department of Pediatrics, University of Colorado Denver, 12800 E 19th Ave, Mail Stop 8302, Aurora, CO 80045, USA

## Abstract

**Background:**

Medulloblastoma is the most common malignant brain tumor in children and remains a therapeutic challenge due to its significant therapy-related morbidity. Polo-like kinase 1 (*PLK1*) is highly expressed in many cancers and regulates critical steps in mitotic progression. Recent studies suggest that targeting PLK1 with small molecule inhibitors is a promising approach to tumor therapy.

**Methods:**

We examined the expression of *PLK1 *mRNA in medulloblastoma tumor samples using microarray analysis. The impact of PLK1 on cell proliferation was evaluated by depleting expression with RNA interference (RNAi) or by inhibiting function with the small molecule inhibitor BI 2536. Colony formation studies were performed to examine the impact of BI 2536 on medulloblastoma cell radiosensitivity. In addition, the impact of depleting *PLK1 *mRNA on tumor-initiating cells was evaluated using tumor sphere assays.

**Results:**

Analysis of gene expression in two independent cohorts revealed that *PLK1 *mRNA is overexpressed in some, but not all, medulloblastoma patient samples when compared to normal cerebellum. Inhibition of PLK1 by RNAi significantly decreased medulloblastoma cell proliferation and clonogenic potential and increased cell apoptosis. Similarly, a low nanomolar concentration of BI 2536, a small molecule inhibitor of PLK1, potently inhibited cell growth, strongly suppressed the colony-forming ability, and increased cellular apoptosis of medulloblastoma cells. Furthermore, BI 2536 pretreatment sensitized medulloblastoma cells to ionizing radiation. Inhibition of PLK1 impaired tumor sphere formation of medulloblastoma cells and decreased the expression of SRY (sex determining region Y)-box 2 (*SOX2*) mRNA in tumor spheres indicating a possible role in targeting tumor inititiating cells.

**Conclusions:**

Our data suggest that targeting PLK1 with small molecule inhibitors, in combination with radiation therapy, is a novel strategy in the treatment of medulloblastoma that warrants further investigation.

## Background

Medulloblastoma is the most common malignant brain tumor in children. While therapy for standard-risk patients has resulted in improved outcomes, high-risk patients do poorly [1]. In addition, there remains significant therapy-related morbidity, particularly in very young patients. Recent genome wide analyses have identified multiple subgroups with differing outcomes [2,3]. These studies show the genetic heterogeneity of medulloblastoma and the need for new therapeutics based on molecular signatures of these tumors. Although a variety of signaling pathways (including Sonic Hedgehog, Wnt and Notch) are known to be associated with medulloblastoma cell biology [4-6], so far new therapeutic interventions based on this knowledge have been slow to develop. Thus, the mainstays of medulloblastoma therapy continue to be surgery, radiation and cytotoxic chemotherapy [7]. While these approaches have improved the outcomes for low-risk patients, those with high-risk disease still have suboptimal outcomes. Furthermore, cranio-spinal radiation treatment itself results in significant long-term morbidity, especially in younger children [8,9], and chemotherapy likewise has major side effects [10]. Thus, there is a critical need for more effective therapies to combat this disease.

To begin to address this need, we examined protein kinase gene expression by transcriptional profiling and found altered expression of multiple protein kinases in medulloblastoma patient samples. Among these kinases is aurora kinase A (AURKA), a target we have recently shown to have therapeutic value in several brain tumors [11,12]. Given that many protein kinases are key regulators of proliferation, invasion, angiogenesis and metastasis, they represent ideal targets for molecularly targeted cancer therapy. Analysis of our previous data suggests that polo-like kinase 1 (*PLK1*) is a potential therapeutic target in medulloblastoma.

PLK1 is essential for mitosis. It promotes mitotic entry by phosphorylating cyclin B1 and CDK1, and it initiates mitotic exit by activating the Anaphase Promoting Complex (APC) [13]. Overexpression of PLK1 promotes chromosome instability and aneuploidy by overriding the G2-M DNA damage and spindle checkpoints [13]. *PLK1 *is overexpressed in a wide variety of cancers, and inhibition of this kinase by shRNA or chemical inhibitors decreases tumor growth both *in vitro *and *in vivo *[13-15]. Importantly, this inhibition preferentially kills cancer cells over normal cells [16,17]. Phase I/II studies of inhibitors of PLK1 in advanced solid tumors in adults have yielded promising results [18,19]. The role of PLK1 in pediatric tumors is less well characterized. Recent studies indicate that it is a target in the treatment of rhabdomyosarcoma and neuroblastoma [14,20,21].

In this study, our goal was to evaluate *PLK1 *as a potential therapeutic target in medulloblastoma. We determined the expression of *PLK1 *mRNA in two independent cohorts of medulloblastoma patients and investigated the effect of PLK1 inhibition by RNA interference (RNAi) and by the small molecule drug BI 2536 on medulloblastoma cells *in vitro*.

## Methods

### Cell lines and primary patient samples

The Daoy and D283 medulloblastoma cell lines were purchased from American Type Cell Culture (Rockville, MD). The ONS-76 medulloblastoma cell line was kindly provided by Dr. James T. Rutka (University of Toronto, Canada). D425 and D458 cell lines were kindly provided by Dr. Darell D. Bigner (Duke University Medical Center, NC). Cell lines were cultured in DMEM (Gibco, Carlsbad, CA) supplemented with 10% fetal bovine serum (Atlanta Biologicals, Lawrenceville, GA).

**Figure 1:**
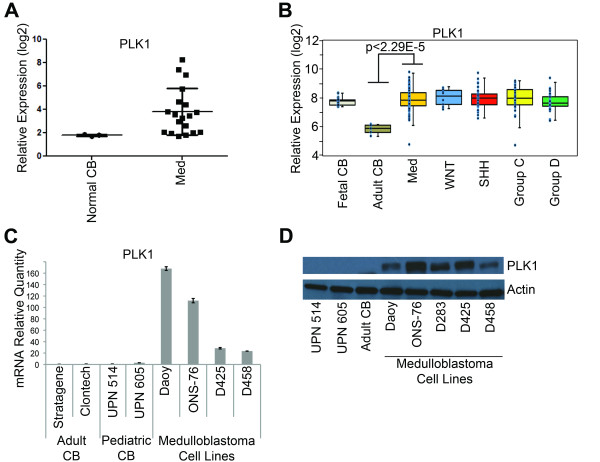
**Overexpression of *PLK1 *in medulloblastoma**. (**A**) *PLK1 *mRNA levels by microarray in 16 primary medulloblastoma (Med) patient samples compared to three normal cerebellum (Normal CB) samples. Error bars represent standard error of the mean (SEM). (**B**) Microarray expression of *PLK1 *mRNA in 120 patients with medulloblastoma (Med). A significant overexpression is seen in medulloblastoma when compared to adult cerebellum (adult CB). There is no significant difference when medulloblastoma samples are divided into four subgroups (WNT, SHH, Group C and Group D). Error bars represent SEM. (**C**) Significantly higher *PLK1 *mRNA expression is seen in medulloblastoma cell lines compared to normal adult cerebellum (Stratagene and Clontech) and pediatric cerebellum (UPN 514 and UPN 605) by qRT-PCR. Error bars represent SEM. (**D**) Western blot of PLK1 protein expression in normal cerebellum and medulloblastoma cell lines

Primary patient samples were obtained from Children's Hospital Colorado and were conducted in accordance with local and federal human research protection guidelines and Institutional Review Board (IRB) regulations. Informed consent was obtained for all specimens collected. Normal brain tissue was collected from autopsy and purchased from Ambion (Austin, TX), Stratagene (Santa Clara, CA) and Clontech Laboratories, Inc. (Mountain View, CA). Normal cerebellar samples in Figure 1 (A, C, D) were obtained from nonmalignant brain biopsies at the Children's Hospital Colorado under IRB guidelines. Normal cerebellar samples UPN 514 and UPN 605 are from 4 year old and 5 year old patients, respectively.

### Gene expression microarray analysis

Sixteen patient tumor samples comprising the first cohort were evaluated for gene expression using Affymetrix U133 Plus 2.0 GeneChip microarrays as previously described [22]. Briefly, samples were collected at the time of surgery and snap-frozen in liquid nitrogen. Ribonucleic acid was extracted from each sample using an RNeasy kit (Qiagen, Valencia, CA) and hybridized to HG-U133 Plus 2.0 GeneChips (Affymetrix, Santa Clara, CA) according to the manufacturer's instructions. Microarray data from the samples was background-corrected and normalized using the gcRMA algorithm. One probe set per gene, based on highest overall expression level across samples, was selected for use in subsequent analyses. Differential expression of genes was determined using a Student's *t*-test [22].

All 120 tumor specimens in the second cohort were obtained in accordance with the Research Ethics Board at the Hospital for Sick Children (Toronto, Canada), as described, and the N. N. Burdenko Neurosurgical Institute (Moscow, Russia) [2]. Gene expression array data were generated and analyzed as described [2].

### Transfection of RNAi vectors

Three siRNAs targeting *PLK1 *mRNA, siPLK1-A (s448), siPLK1-B (s449) and siPLK1-C (s450), and a non-targeting siRNA were transfected into medulloblastoma cell lines using the siPORT NeoFX Transfection Agent (Ambion). A final concentration of 5 nM of siRNA was transfected into the medulloblastoma cell lines. The manufacturer's suggested protocol for a reverse transfection was used with the siRNA. Of the three siRNAs targeting *PLK1*, siPLK1-A was used in further experiments due to its higher efficacy in inhibiting *PLK1 *(Additional file 1: Figure S1 [B, C, D]).

Two shRNA vectors targeting *PLK1 *mRNA (1073: CCGGCGAGCTGCTTAATGACGAGTTCTCGAGAACTCGTCATTAAGCAGCTCGTTTTTG and 1325: CCGGCCCGAGGTGCTGAGCAAGAAACTCGAGTTTCTTGCTCAGCACCTCGGGTTTTTG) and a non-targeting shRNA (shNTC) were purchased from the Functional Genomics Facility at the University of Colorado, Boulder, and transfected into medulloblastoma cell lines using the Lipofectamine 2000 Transfection Reagent (Invitrogen, Carlsbad, CA). The shPLK1 1325 vector was used in further experiments due to its greater inhibition of *PLK1 *(Additional file 1: Figure S1 [A, D]). One microgram of shRNA for a 6-well plate was transfected into the medulloblastoma cell lines. The ratio used for the forward transfections was 1 microgram of shRNA DNA: 2 μl of Lipofectamine 2000 Transfection Reagent.

### Quantitative real-time polymerase chain reaction

Ribonucleic acid was isolated 48 hours after transfection using a Qiagen RNeasy kit (Valencia, CA). TaqMan gene expression primers and probes for *PLK1 *(Hs00153444_m1), *SOX2 *(Hs01053049_s1), *NES *(Hs00707120_s1), *Nanog *(Hs002387400_g1), *c-Myc *(Hs01570247_m1), and *GAPDH *(Hs99999905_m1) were purchased from Applied Biosystems (Carlsbad, CA). Assays were performed in triplicate according to the manufacturer's recommendations. *GAPDH *was used as an endogenous control and the gene expression relative quantity was calculated using the ΔΔC_t _method. Gene expression assays were performed on an ABI StepOnePlus Real-Time PCR system.

### Small molecule inhibitors of PLK1

The small molecule PLK1 inhibitors BI 2536 and BI 6727 were purchased from Axon Medchem (Groninberg, Netherlands). The drugs were reconstituted in dimethyl sulfoxide (DMSO) and aliquots were stored in a desiccator at -20°C. An equivalent amount of DMSO for the highest concentration of drug was used for each experiment as a vehicle control.

### Cell proliferation and apoptosis assays

Cell proliferation was determined by MTS [3-(4, 5-dimethylthiazol-2-yl)-5-(3-carboxymethoxyphenyl)-2-(4-sulfophenyl)-2H-tetrazolium] assay using CellTiter 96 AQueous One Solution (Promega, Madison, WI). Seventy-two hours after transfection with siRNA, 20 μl of MTS reagent was added to the wells already containing 100 μl of media. For drug treatment, cells were plated for 24 hours before adding BI 2536 or BI 6727. Then 72 hours after the addition of the drug, 30 μl of MTS reagent were added to the wells to make a final volume of 180 μl. Plates were read using a BioTek Synergy 2 plate reader (Winooski, VT) every hour for 4 hours after the addition of the MTS reagent. Experiments were done in triplicate and background absorbance was subtracted from all wells before analysis.

For the colony formation assay, cells were transfected with shRNA for 48 hours and then plated at 500 cells per well of a 6-well plate in triplicate. For drug treatment, 500 cells per well of a 6-well plate were plated in triplicate 24 hours before addition of BI 2536. Wells were then treated with drug for 24 hours and then allowed to grow in normal culture medium. After seven days of growth, the medium was aspirated, the wells were washed with PBS, and the colonies were stained with 0.5% crystal violet/25% methanol solution. The number of colonies per well was counted using a dissecting microscope with a threshold of 50 cells necessary to constitute a colony.

Apoptosis was assessed 72 hours after siRNA transfection or after 24 hours of BI 2536 treatment followed by 24 hours in normal culture medium. Cells were counted following staining with Guava ViaCount reagent (Millipore, Billerica, MA) and the amount of apoptosis determined using Guava Nexin reagent (Millipore). Samples were run on a Guava EasyCyte Plus flow cytometer (Millipore).

### Western blotting

Protein lysates were obtained from samples using RIPA buffer (Thermo Scientific, Rockford, IL) with protease inhibitors added. Western blotting was performed per standard methods. Antibodies for PLK1 (#4535) and Actin (MAB1501) were purchased from Cell Signaling Technology (Danvers, MA) and Millipore, respectively. Secondary antibodies conjugated to horseradish-peroxidase were used in conjunction with a chemiluminescent reagent to visualize protein bands.

### Combination of BI 2536 and ionizing radiation

Five hundred cells per well of a 6-well plate were plated in triplicate for 24 hours before addition of BI 2536. Cells were exposed to drug for 24 hours, and then drug-containing medium was aspirated and normal culture medium was added. Cells were then immediately irradiated. After eight days of additional growth, wells were stained with crystal violet solution and colonies were counted as described above. Survival curves were generated after normalizing for the amount of BI 2536-induced death. Non-linear regressions were calculated for each line. The radiation dose intersecting the non-linear regression for a 10% (SF0.1) and 50% (SF0.5) surviving fraction was calculated for each drug dose. The sensitizer enhancement ratio (SER) was then calculated as follows:

$$\text{SER}=\frac{\text{S}{\text{F}}_{\text{x}}\text{for DMSO}}{\text{S}{\text{F}}_{\text{x}}\text{for }\times \text{ nM BI 2536}}$$

### Tumor sphere formation

Daoy medulloblastoma cells were either transfected with shRNA or treated with 5 nM BI 2536. Forty-eight hours after transfection or drug treatment, cells were trypsinized and counted. Ten thousand cells were seeded in a low-attachment 6-well plate in neurobasal medium (Gibco) supplemented with fibroblast growth factor, 20 ng/mL (Sigma-Aldrich, St. Louis, MO), B-27 (Gibco), epidermal growth factor, 20 ng/mL (Sigma-Aldrich), and L-glutamine (Gibco). Tumor spheres were allowed to grow for 7 days. After 7 days, pictures were taken with an inverted microscope and subsequently the tumor spheres were either processed for RNA isolation, or dissociated and passaged to form secondary tumor spheres. The primary tumor spheres were dissociated with Accutase (Millipore, Scr005) and resuspended in PBS. Ten thousand cells from the primary tumor spheres were seeded on a low attachment plate. The formation of secondary tumor spheres was seen 4 days after seeding. The diameter of the tumor spheres was measured using QCapture Pro software from saved images captured at 4× maginification.

### Statistical analysis

Statistical significance was determined using a Student's *t*-test. Error bars represent the standard error of the mean (n ≥ 3). GraphPad Prism 5 was used to calculate IC_50 _values and to compute the nonlinear regression equations.

## Results

### Plk1 is overexpressed in medulloblastoma

We initially hypothesized that kinases involved in cell cycle regulation would be likely candidates as novel therapeutic targets in medulloblastoma. To begin to address this question, we first examined expression of cell cycle-regulated kinases in a cohort of sixteen medulloblastoma patients we had previously studied [23]. We found that expression of *PLK1 *mRNA was altered in most, but not all, of our patient samples when compared to normal cerebellum (Figure 1A). There was no clear correlation between the high *PLK1*-expressing samples and age, gender or outcomes. Recent genomic analysis defined four major subgroups of medulloblastoma. The 4 major subgroups are Sonic Hedgehog signaling (SHH), Wnt signaling (WNT), Group C and Group D. Group C and D tend to be more aggressive than the SHH or WNT signaling groups [2]. To further elucidate whether there was a correlation within the subgroups of medulloblastoma, we examined expression of *PLK1 *mRNA in a cohort of 120 recently described medulloblastoma samples [2]. Medulloblastoma samples expressed significantly higher *PLK1 *mRNA compared to adult cerebellum (p < 0.00003), but *PLK1 *mRNA expression was not significantly higher when compared to fetal cerebellum (Figure 1B). When examined at the genomic subgroup level, there was no difference in *PLK1 *mRNA expression among the four major genomic subgroups (Figure 1B). These data indicate that *PLK1 *may be associated with the oncogenic process in general and is not specific to a particular molecular subgroup of medulloblastoma. We next evaluated expression of *PLK1 *mRNA in a panel of well-characterized medulloblastoma cell lines. Consistent with our patient tissue data, all medulloblastoma cell lines tested expressed *PLK1 *mRNA at significantly higher levels (p < 0.01) compared to normal pediatric and adult cerebellum (Figure 1C). We next examined PLK1 protein expression in normal pediatric and adult cerebellum as well as a panel of medulloblastoma cell lines. As seen in Figure 1D, pediatric cerebellum (UPN 514 and 605) and adult cerebellum have minimal PLK1 protein expression while all the medulloblastoma cell lines have increased but varied levels of PLK1 protein.

### Inhibition of PLK1 suppresses medulloblastoma cell growth and colony forming ability

**Figure 2:**
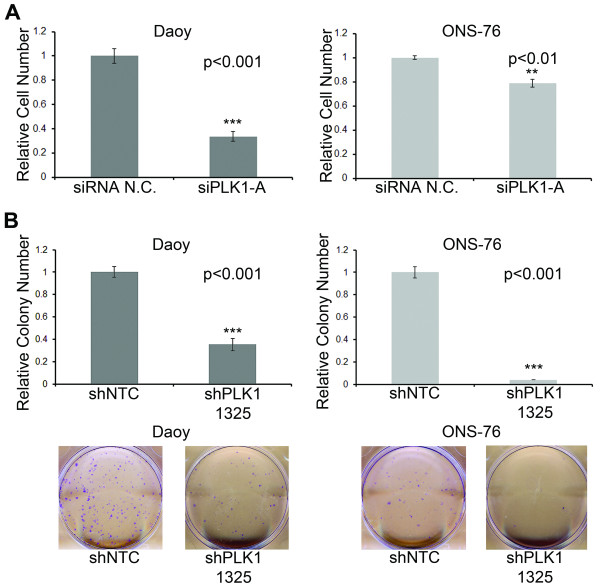
**Decrease in medulloblastoma cell growth and colony forming ability following *PLK1 *mRNA inhibition by RNAi**. (**A**) A significant decrease in relative cell number was seen in both Daoy and ONS-76 cell lines by MTS assay 72 hours following siRNA-mediated inhibition of *PLK1 *mRNA. Error bars represent SEM. (**B**) Long-term decrease in growth as seen through a colony formation assay following knockdown of *PLK1 *mRNA with shRNA in Daoy and ONS-76 cell lines. Representative pictures of wells for each cell line are below the quantifying graphs. Error bars represent SEM

To examine whether PLK1 was functionally important for medulloblastoma tumorigenesis, we initially decreased expression of *PLK1 *mRNA by siRNA and shRNA. Knockdown of *PLK1 *was verified by qRT-PCR and by western blot analysis (Additional file 1: Figure S1). For further experiments siPLK1-A and shPLK1 1325 were used due to their greater inhibition of PLK1. Using the MTS assay, we found a significant decrease in cell proliferation of two different medulloblastoma cell lines, Daoy and ONS-76, transfected with *PLK1 *siRNA compared to control siRNA (Figure 2A). We chose to evaluate Daoy and ONS-76 cells because they are well characterized, have high levels of *PLK1 *mRNA and we have previous experience transfecting them [24]. Importantly, Daoy is p53 mutant and ONS-76 has wild type p53 so any potential effect of p53 status on PLK1 inhibition can be interesting. To evaluate a longer-term impact, we then performed colony formation assays on cells transfected with control or *PLK1 *shRNA. Once again, inhibition of *PLK1 *mRNA by RNAi significantly decreased medulloblastoma cell growth as measured by their ability to form colonies (Figure 2B). Interestingly, in ONS-76 cells there was a much more marked inhibition in the colony formation capability compared to measuring just cell proliferation (89% inhibition versus 20% inhibition, respectively). These data may reflect the impact of PLK1 on tumor cell self-renewal compared to just mitosis.

### PLK1 inhibition induces apoptosis in medulloblastoma cells

**Figure 3:**
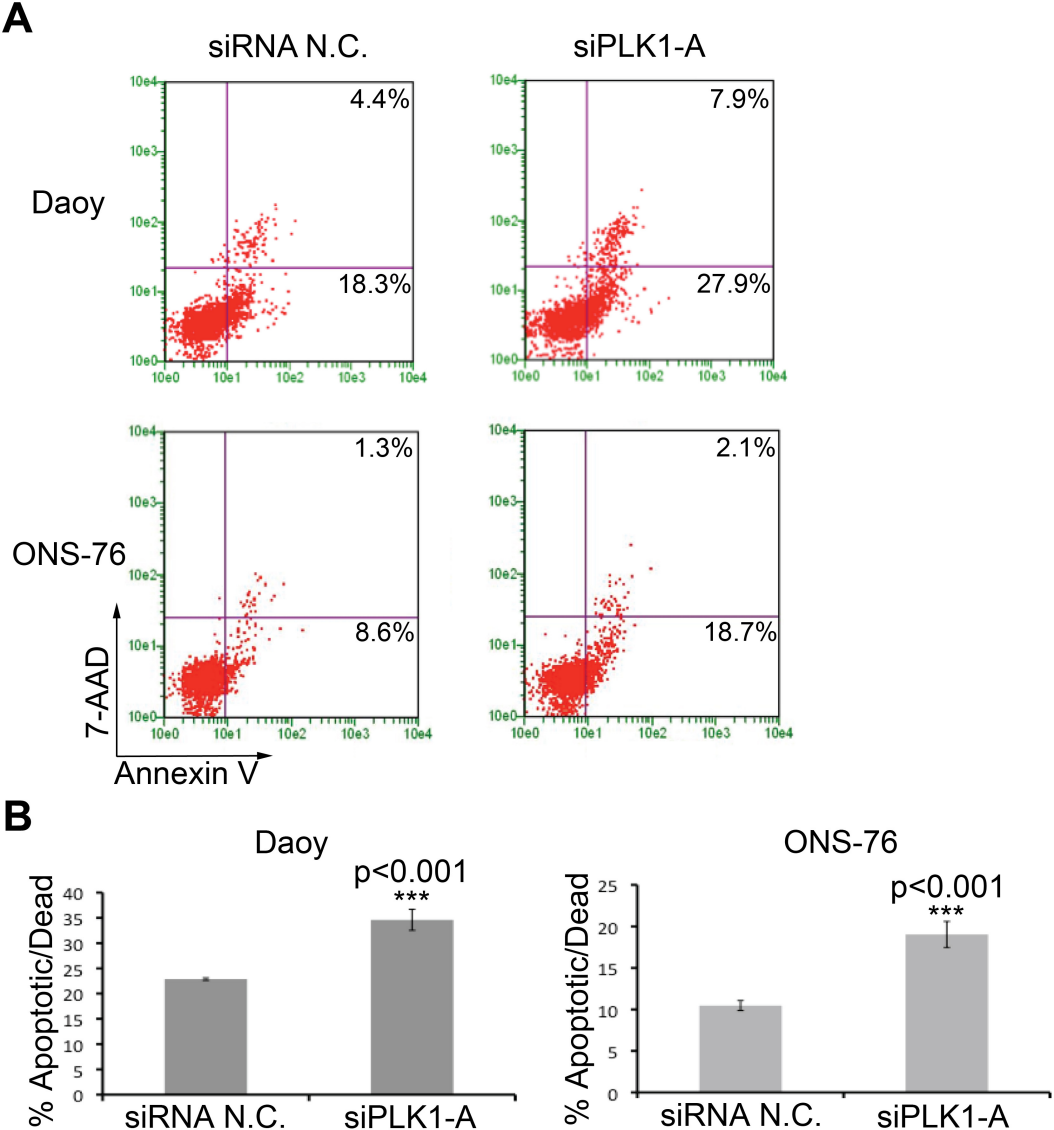
**Increased apoptosis following *PLK1 *mRNA inhibition by RNAi**. (**A**) Medulloblastoma cell lines Daoy and ONS-76 were transfected with either a non-silencing siRNA (siRNA N.C.) or a siRNA targeting PLK1 (siPLK1-A) for 72 hours and stained with Guava Nexin reagent. Representative flow cytometry plots for the two cell lines are shown. Percentages of cells in the bottom left quadrant are live while cells to the right of the vertical line are apoptotic or dead. (**B**) Quantifying bar graphs showing a significant increase in the percentage of apoptotic/dead cells following *PLK1 *mRNA knockdown with siRNA in both Daoy and ONS-76 cell lines. Error bars represent SEM

Inhibition of PLK1 induces the spindle checkpoint with subsequent induction of cell death [25]. To evaluate whether decreased expression of *PLK1 *mRNA resulted in apoptosis in medulloblastoma, we analyzed Annexin V expression on medulloblastoma cells by flow cytometry. As shown in the representative plots in Figure 3A, siPLK1-A, but not non-silencing control (siRNA N.C.), potently induced apoptosis as detected by increased Annexin V expression in both Daoy and ONS-76 medulloblastoma cell lines. The difference in apoptosis between the non-silencing siRNA control and siPLK1-A was statistically significant in multiple independent replicates (Figure 3B). These data suggest that *PLK1 *could indeed be a promising therapeutic target for medulloblastoma.

### Small molecule inhibitor BI 2536 is a potent inhibitor of medulloblastoma cell growth by increased apoptosis

**Figure 4:**
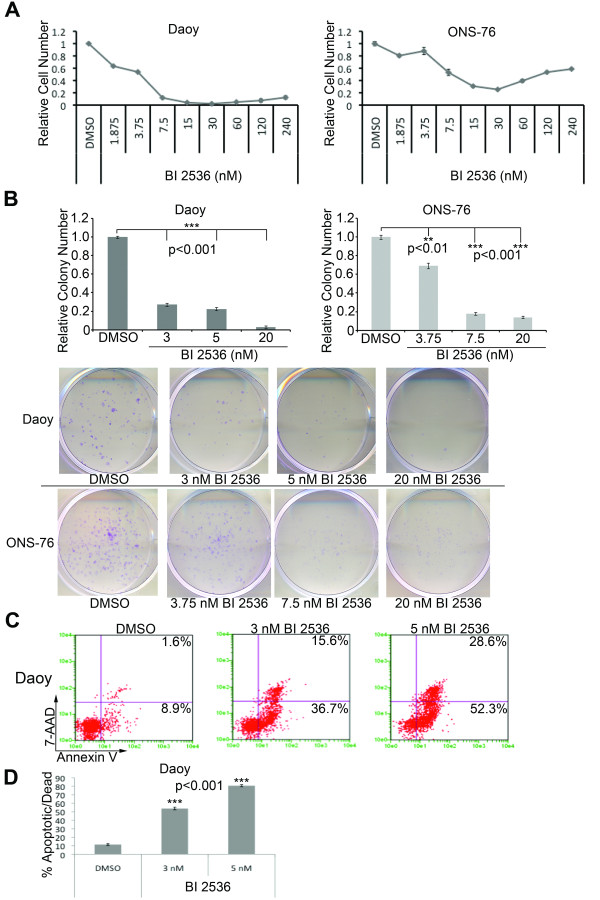
**Inhibition of PLK1 with BI 2536 decreases cell growth and increases apoptosis**. (**A**) Decrease in the relative cell number of two medulloblastoma cell lines, Daoy and ONS-76, through a wide range of BI 2536 concentrations treated for 72 hours. Error bars represent SEM. (**B**) Quantifying bar graph showing a significant decrease in the ability of two medulloblastoma cell lines in forming colonies following 24 hour treatment with BI 2536. Error bars represent SEM. Shown below are representative pictures of the wells from the colony formation assay. (**C**) Representative flow cytometry plots for Daoy cells stained with Guava Nexin reagent following 24 hours of treatment with BI 2536. Percentages of cells in the bottom left quadrant are live while cells to the right of the vertical line are apoptotic or dead. (**D**) Bar graph quantifying the percentage of apoptotic/dead cells following Guava Nexin reagent staining. Error bars represent SEM

Recently, several inhibitors of PLK1 have been described [13]. Among these is the dihydropteridinone derivative BI 2536 (Boehringer Ingelheim, Ingelheim, Germany). We determined whether BI 2536, like *PLK1 *siRNA, decreases proliferation of medulloblastoma cells. Daoy and ONS-76 cells were treated with varying concentrations of BI 2536 and cell proliferation was evaluated by the MTS assay. BI 2536 potently inhibited cell growth with an IC_50 _of 5 nM for Daoy and 7.5 nM for ONS-76 cells after 72 hours of treatment (Figure 4A). To further examine the effects of BI 2536 on medulloblastoma cells, we performed colony formation assays. We found that BI 2536 strongly suppressed the ability of medulloblastoma cells to form colonies. Representative colonies are shown in Figure 4B.

To determine whether this decreased proliferation was due to apoptosis, we evaluated Annexin V expression on the surface of BI 2536-treated medulloblastoma cells by flow cytometry. Representative plots are shown in Figure 4C for Daoy and in Additional file 2: Figure S2A for ONS-76. Annexin V positive--7-aminoactinomycin D (7-AAD) negative cells, indicative of early apoptosis, were present at low levels in DMSO control-treated cells. This population increased with escalating doses of BI 2536. In addition, the Annexin V positive--7-AAD positive population was significantly enhanced in BI 2536 cells, indicating increased late apoptosis. The total percentage of apoptosis is quantified in Figure 4D and in Additional file 2: Figure S2B for Daoy and ONS-76, respectively.

### BI 2536 enhances radiation sensitivity of medulloblastoma cells

**Figure 5:**
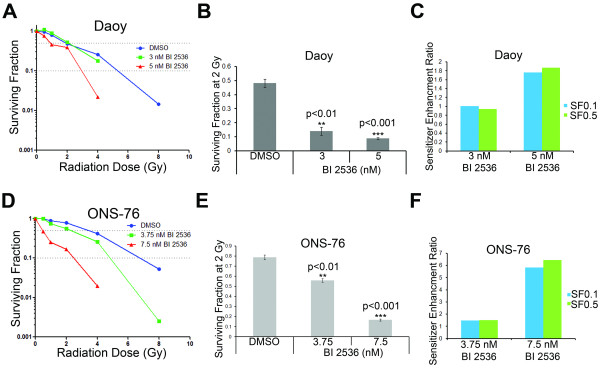
**Pretreatment with BI 2536 enhances radiation sensitivity in medulloblastoma cell lines**. (**A**) Line graph plotting the surviving fraction of Daoy cells given different radiation doses following 24 hours of treatment with BI 2536 or the vehicle control DMSO. Blue line and symbols represent DMSO treated cells. Green line and symbols represent cells treated with 3 nM BI 2536. Red line and symbols represent cells treated with 5 nM BI 2536. (**B**) Bar graph showing the quantification of the surviving fraction following 2 Gray (Gy) irradiation. (**C**) Sensitizer enhancement ratio (SER) for Daoy cells. Blue bars are calculated using a survival fraction of 10% (SF0.1) and green bars are calculated using a survival fraction of 50% (SF0.5). (**D**) Line graph plotting the surviving fraction of ONS-76 cells. Blue line and symbols represent DMSO treated cells. Green line and symbols represent cells treated with 3.75 nM BI 2536. Red line and symbols represent cells treated with 7.5 nM BI 2536. (**E**) Bar graph quantifying the surviving fraction at the 2 Gray (Gy) level of irradiation for ONS-76 cells. Error bars represent SEM. (**F**) Bar graph depicting the sensitizer enhancement ratio in ONS-76 cells. Blue bars are calculated using a survival fraction of 10% (SF0.1) and green bars are calculated using a survival fraction of 50% (SF0.5)

BI 2536 is a promising agent for combination therapy in medulloblastoma. To investigate whether BI 2536 enhances cellular sensitivity to ionizing radiation, medulloblastoma cells were exposed to BI 2536 for 24 hours before irradiation and the effects evaluated using the colony formation assay. The results show that the survival fractions (SF) at different radiation dosages were reduced in Daoy (Figure 5A) and ONS-76 (Figure 5D) cells after they were exposed to BI 2536. Survival fractions of BI 2536-pretreated cells after 2 Gray irradiation were significantly lower than those of untreated cells (Figure 5 [B, E]). Nonlinear regressions were fit to the curves to assist in calculating the sensitizer enhancement ratio (SER) (Additional file 3: Figure S3). The sensitizer enhancement ratios were 1.8 for Daoy at 10% cell survival (SF0.1) and 1.9 at 50% cell survival (SF0.5) with 5 nM BI 2536 pre-treatment (Figure 5C). For ONS-76 cells pretreated with 7.5 nM BI 2536, the SERs were 5.8 and 6.4 for SF0.1 and SF0.5, respectively (Figure 5F). Thus, the radiation survival curves obtained by the colony formation assay showed that BI 2536 pretreatment sensitized human medulloblastoma cells to ionizing radiation.

### Inhibition of PLK1 mRNA decreases tumor sphere formation and decreases SOX2 mRNA expression

**Figure 6:**
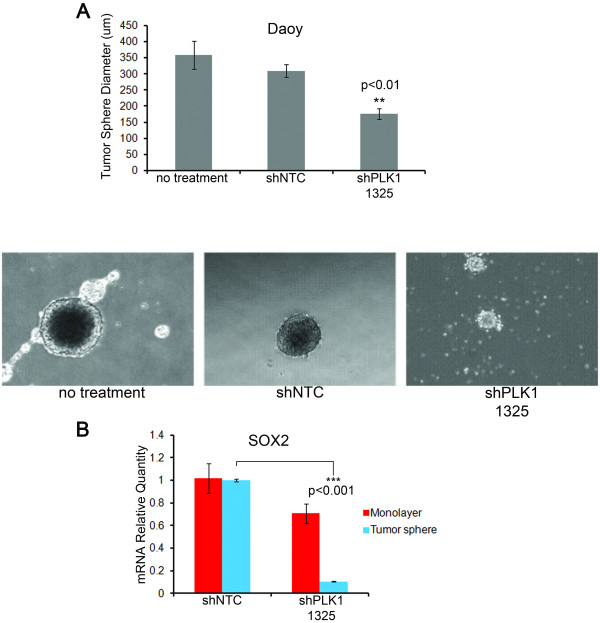
**Reduction in both tumor sphere size and *SOX2 *expression following *PLK1 *mRNA inhibition by RNAi**. (**A**) Bar graph quantifies the average tumor sphere diameter for the three different conditions: Daoy undergoing no treatment, transfected with non-targeting shRNA (shNTC), or shRNA targeting PLK1 (shPLK1 1325). Error bars represent SEM. Shown below the bar graph are the representative pictures of Daoy primary tumor spheres. The diameter of the tumor spheres was measured using QCapture Pro software from saved images. Magnification 4×. (**B**) Bar graph quantifying the levels of *SOX2 *mRNA expression in Daoy cells following shRNA transfection. Red bars represent mRNA levels in cells grown in standard adherent monolayer conditions. Blue bars depict mRNA levels for cells grown in the non-adherent tumor sphere conditions. Error bars represent SEM

We next evaluated the hypothesis that inhibition of *PLK1 *mRNA impairs tumor initiating cells in medulloblastoma. We cultured Daoy cells in a non-adherent culture system in the presence of serum-free stem cell media (neurobasal with EGF, FGF and B27). Under these conditions Daoy cells form robust tumor spheres (Figure 6A). These tumor spheres upregulate mRNA expression of many stem and progenitor markers including *SOX2, Nanog, NES *and *c-Myc *as shown in Additional file 4: Figure S4A and as previously described for CD133 and CD44 [26]. Daoy cells were transfected with non-targeting shRNA (shNTC) or with shPLK1 plasmids. Forty-eight hours post-transfection, cells were transferred to the non-adherent culture system and tumor sphere growth was monitored. By seven days post-transfection, untreated and shNTC-transfected cells formed tumor spheres with a mean diameter of 300-360 μm. In contrast, shPLK1-transfected cells formed significantly smaller (176 μm) spheres (Figure 6A). We next measured a panel of stem/progenitor cell markers by qRT-PCR (Additional file 4: Figure S4B). We found that RNAi-mediated inhibition of *PLK1 *mRNA in tumor spheres significantly decreased *SOX2 *mRNA expression compared to control transfected cells (Figure 6B) with no significant change in *NES, Nanog*, and *c-Myc *mRNA expression (Additional file 4: Figure S4B). Interestingly, inhibition of *PLK1 *mRNA in standard adherent cultures did not significantly alter *SOX2 *mRNA expression (Figure 6B). The mechanism by which PLK1 acts upon SOX2 is unclear and requires further detailed study.

We then analyzed the impact of BI 2536 on tumor sphere formation in the Daoy cell line. BI 2536 decreased the size of the tumor spheres (479.2 μm for the DMSO control treated vs. 142 μm for the 5 nM BI 2536 treated) consistent with the RNAi experiments (Additional file 5: Figure S5A). Interestingly, if we dissociate these primary tumor spheres and reseed them, the diameter of the serially passaged secondary tumor spheres is also significantly impaired (396.5 μm for the DMSO control treated vs. 171.4 μm for the cells previously treated with 5 nM BI 2536; Additional file 5: Figure S5B). We conclude therefore that PLK1 plays a key role in regulating stem-like characteristics of tumor cells.

## Discussion

Therapy-associated side effects in medulloblastoma have led to a concentrated search for novel therapeutic targets, particularly targets for which inhibition has radiosensitizing potential and minimal toxicities. Recent genomic studies have begun to unravel the molecular mechanisms involved in medulloblastoma, but have not yet resulted in novel therapeutic agents [2,3,27]. Analysis of protein kinase gene expression revealed that expression of multiple protein kinases was altered in medulloblastoma, including several components of the mitotic machinery such as aurora kinase A and *PLK1 *[23]. Perturbing mitosis by disrupting the proper formation of mitotic spindles required for chromosome alignment and segregation has been shown to preferentially kill cancer cells [28].

It is well established that *PLK1 *plays an important role in cell cycle regulation by functioning in centrosome maturation, spindle formation, mitotic entry and cytokinesis. Elevated *PLK1 *levels have been found in many adult cancers, including breast and colorectal cancer, and in pediatric cancers, including neuroblastoma and rhabdomyosarcoma [20,21,29]. While *PLK1 *mRNA expression is upregulated in medulloblastoma, the significance of *PLK1 *in the pathogenesis and management of this pediatric brain tumor is not well understood.

In this study we demonstrate that *PLK1 *mRNA is overexpressed in two independent medulloblastoma cohorts when compared to normal cerebellum. Of note, fetal tissues expressed very high levels of *PLK1 *mRNA compared to adult brain tissues. This may reflect the critical role *PLK1 *in regulating mitosis. Indeed PLK1 is essential for progression into mitosis during embryonic development. *PLK1*-deficient cells displayed mitotic infidelity resulting in mitotic arrest and finally death during zebrafish embryogenesis [30]. Furthermore, *PLK1 *homozygous null mice were found to be embryonic lethal and the incidence of tumors in *PLK1 *heterozygotes was three-fold greater than that in their wild-type counterparts, again emphasizing the importance of *PLK1 *in normal embryogenesis and development [31]. Interestingly, not all tumor samples overexpressed *PLK1 *mRNA, further emphasizing the molecular heterogeneity of this tumor.

Decreasing the expression of *PLK1 *mRNA by RNAi clearly resulted in growth suppression and induction of apoptosis in medulloblastoma cells. Furthermore, we show that inhibition of PLK1 by a small molecule inhibitor, BI 2536, results in a significant reduction in the proliferation of medulloblastoma cells both in short-term and long-term assays. Importantly, IC_50 _values were in the low nanomolar range, which is in line with achievable therapeutic plasma concentrations demonstrated in clinical phase I/II trials of BI 2536 [18]. Treatment with 5 nM BI 2536 in Daoy and 7.5 nM in ONS-76 medulloblatoma cells induced apoptosis, which is consistent with results found in other cancer cells [20].

BI 2536 (Boehringer Ingelheim, Ingelheim, Germany) is a first-in-class PLK1 inhibitor. Not only is it an ATP-competitive kinase inhibitor that inhibits the enzymatic activity of PLK1, it also shows over 1,000-fold selectivity for PLK1 against a large panel of other tyrosine and serine/threonine kinases [18,29]. In dose-escalation Phase I trials, BI 2536 was well tolerated [18]. Several Phase II studies are underway or have recently been completed for BI 2536 [29]. In addition to BI 2536, there are several other inhibitors of PLK1 in development and undergoing clinical testing. These include BI 6727 (Boehringer Ingelheim, Ingelheim, Germany), GSK461364 (GlaxoSmithKline, Middlesex, UK) and HMN-214 (Nippon Shinyaku Co. Ltd, Kyoto, Japan). However, there are currently no clinical studies of PLK1 inhibitors in any pediatric cancers. Our data and those of Ackermann, et al., and Hu, et al., strongly argue for development of such studies in pediatric solid tumors [20,21]. In particular our data show that PLK1 is a target in all subgroups of medulloblastomas making it ideal for clinical trials.

Radiation is a key component of medulloblastoma therapy. Unfortunately, it results in significant morbidity, particularly in very young patients [9]. Thus, agents that radiosensitize medulloblastoma cells would be of great utility. Here we show that low nanomolar concentrations of BI 2536 strongly decreased the surviving fraction of tumor cells in response to radiation and increased the sensitizer enhancement ratios. These results indicate that BI 2536 can effectively enhance medulloblastoma cell radiosensitivity *in vitro*. These data are in accordance with previous studies showing increased radiosensitivity in malignant cells depleted of *PLK1 *mRNA by RNAi [32].

It has been hypothesized that medulloblastoma tumors contain stem cell-like tumor-initiating cells that are more resistant to therapy [33,34]. Here we found that inhibition of *PLK1 *expression significantly decreased the tumor sphere size and decreased the expression of the stem cell marker *SOX2*. Interestingly, the decrease in stem cell markers was more pronounced in tumor spheres than in monolayer cells cultured in normal adherent conditions. Thus, there is a clear role for *PLK1 *in tumor-initiating cells, a finding hinted at in recent data from neuroblastoma [14]. It will be important to elucidate in detail the specific mechanisms by which *PLK1 *mediates tumor-initiating cell growth.

In total, our data make a strong argument for further exploring the role of PLK1 inhibition in medulloblastoma. Regarding the PLK1 inhibitors, a second PLK1 inhibitor, BI 6727, has been used in several studies [35]. BI 6727 is similar to BI 2536 in its *in vitro *activity in Daoy and ONS-76 medulloblastoma cell lines (Additional file 6: Figure S6). In *in vivo *studies, BI 6727 shows better toxicity and pharmacokinetic profile compared to BI 2536. The next step will be to perform carefully constructed animal studies. We plan to perform orthotopic cerebellar xenograft *in vivo *studies with BI 6727. We will especially take in to consideration dosing schedules and pharmacodynamic marker evaluation. These data will assist in developing future clinical trials.

## Conclusion

Our data, together with previous studies, strongly suggest that targeting PLK1 with small molecule inhibitors in combination with radiation therapy is both a novel strategy in the treatment of medulloblastoma and one that warrants further study.

## Competing interests

The authors declare that they have no competing interests.

## Authors' contributions

PSH performed the cell studies and helped draft the manuscript. SV performed the tumor sphere studies, western blot analysis and helped edit the manuscript. IA performed the qRT-PCR and western blot analysis and helped perform the clonogenic assays. JK assisted in the sensitizer enhancement ratio studies. DKB and AMD performed tissue and microarray analysis of cohort 1 and AD and MDT performed tissue and microarray analysis of cohort 2. MHH and NKF collected tissue, helped convive the study and edited the manuscript. RV conceived and supervised the study and drafted the original manuscript. All authors approved the manuscript.

## Pre-publication history

The pre-publication history for this paper can be accessed here:

http://www.biomedcentral.com/1471-2407/12/80/prepub

## Supplementary Material

Additional file 1 **Figure S1**. RNAi transfection decreases *PLK1 *mRNA and protein levels in medulloblastoma cell lines. Quantifying bar graphs depicting significant decreases in *PLK1 *mRNA levels in Daoy medulloblastoma cell line transfected: (A) with either a non-targeting shRNA (shNTC) or two different shRNAs targeting PLK1 (shPLK1 1073 and shPLK1 1325), (B) with either a scrambled siRNA (siRNA N.C.) or three different siRNAs targeting PLK1 (siPLK1-A, siPLK1-B, and siPLK1-C). Error bars represent SEM. (C) ONS-76 cells show a significant decrease in PLK1 mRNA expression following transfection with siPLK1-A. Error bars represent SEM. (B) Western blot analysis confirms that RNAi-mediated knockdown of PLK1 decreases the level of PLK1 protein in both Daoy and ONS-76 cells. Click here for file

Additional file 2 **Figure S2**. Inhibition of PLK1 by the small molecule inhibitor BI 2536 increases apoptosis in ONS-76 cells. (A) Representative plots for ONS-76 cells stained with Guava Nexin reagent following 24 hours of treatment with BI 2536 or an equivalent amount of the drug's vehicle DMSO. Percentages of cells in the bottom left quadrant are live while cells to the right of the vertical line are apoptotic or dead. (B) The bar graph quantifies the average percentage of apoptotic/dead cells. Error bars represent SEM. Click here for file

Additional file 3 **Figure S3**. Nonlinear regression lines for medulloblastoma cells treated with BI 2536 followed by radiation. (A) Daoy cells: the blue line is for DMSO treated cells, the green line for cells treated with 3 nM BI 2536, and the red line for cells treated with 5 nM BI 2536. (B) ONS-76 cells: the blue line is for DMSO treated cells, the green line is for cells treated with 3.75 nM BI 2536, and the red line is for cells treated with 7.5 nM BI 2536. The R^2 ^values for each line are listed below their respective graphs. Click here for file

Additional file 4 **Figure S4**. Quantitative real-time PCR measure of the mRNA expression of stem/progenitor markers in Daoy cells. (A) Daoy cells were cultured under standard conditions or serum-free conditions. Stem/progenitor cell markers increase when cells are grown under serum-free tumor sphere conditions compared to standard serum containing adherent monolayer conditions. (B) Knockdown of *PLK1 *mRNA with shPLK1 1325 decreases *SOX2 *mRNA in Daoy cells grown as tumor spheres. There was no significant change in *Nanog, NES*, or *c-Myc *mRNA. Click here for file

Additional file 5 **Figure S5**. BI 2536 decreases Daoy tumor sphere diameter. Daoy cells treated for 48 hours with 5 nM BI 2536 show decreased tumor sphere diameter in both (A) primary tumor spheres and (B) secondary tumor spheres. The diameter of the tumor spheres was measured using QCapture Pro software from saved images. Magnification 4×. Click here for file

Additional file 6 **Figure S6**. Second generation PLK1 inhibitor BI 6727 induces growth arrest of medulloblastoma similar to BI 2536. Decrease in the relative cell number of two medulloblastoma cell lines, (A) Daoy and (B) ONS-76, treated for 72 hours with either BI 2536 (blue line and symbols) or BI 6727 (green line and symbols) as measured by MTS assay. Error bars represent SEM. Click here for file
