# Supplementary figures and images for: Polo-like kinase 1 (PLK1) inhibition suppresses cell growth and enhances radiation sensitivity in medulloblastoma cells
Source: BMC Cancer. 2012 Mar 5;12:80. doi: 10.1186/1471-2407-12-80 (PMC3311601; doi:10.1186/1471-2407-12-80)

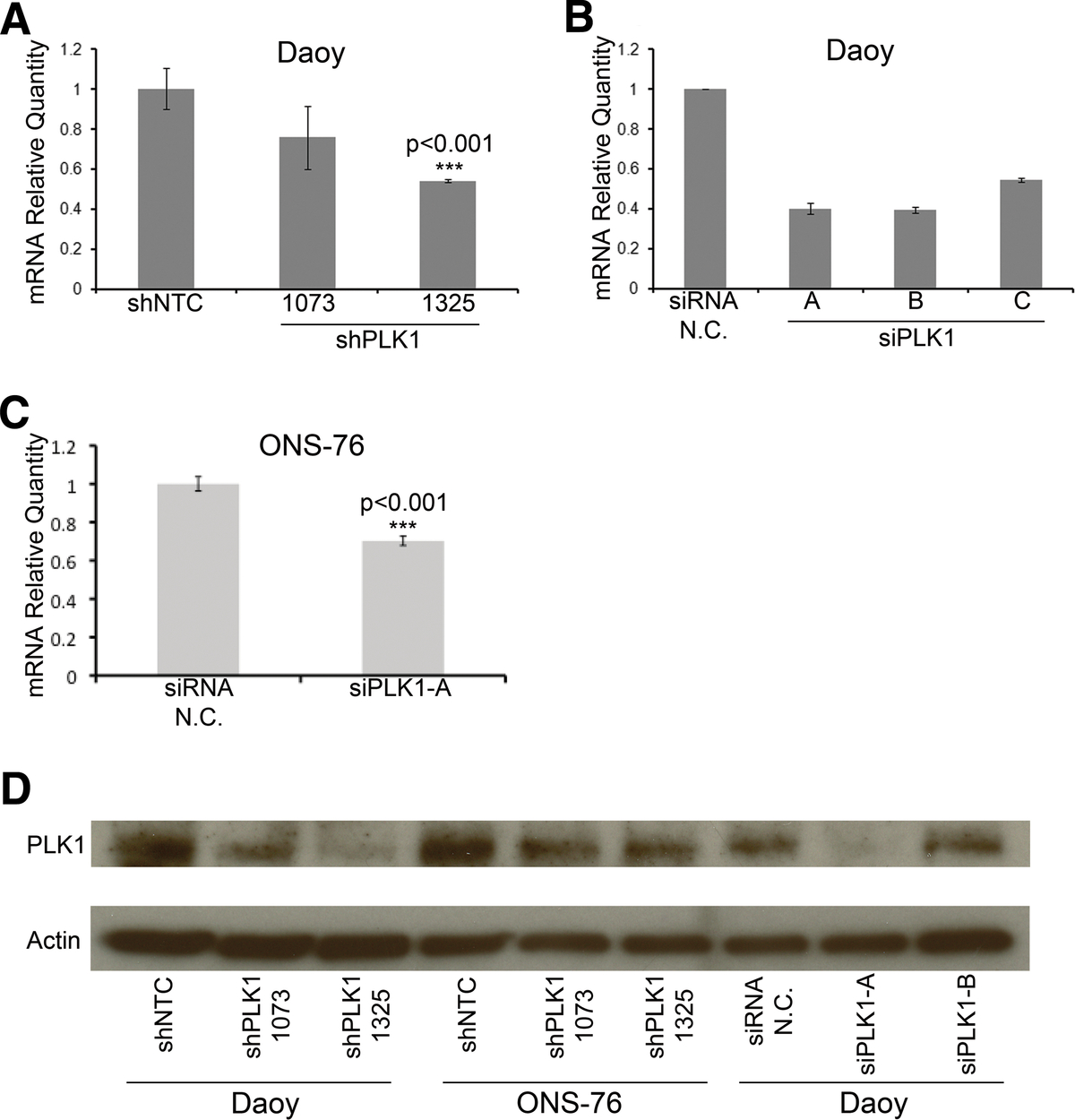

Supplement: Additional file 1 — Figure S1. RNAi transfection decreases PLK1 mRNA and protein levels in medulloblastoma cell lines. Quantifying bar graphs depicting significant decreases in PLK1 mRNA levels in Daoy medulloblastoma cell line transfected: (A) with either a non-targeting shRNA (shNTC) or two different shRNAs targeting PLK1 (shPLK1 1073 and shPLK1 1325), (B) with either a scrambled siRNA (siRNA N.C.) or three different siRNAs targeting PLK1 (siPLK1-A, siPLK1-B, and siPLK1-C). Error bars represent SEM. (C) ONS-76 cells show a significant decrease in PLK1 mRNA expression following transfection with siPLK1-A. Error bars represent SEM. (B) Western blot analysis confirms that RNAi-mediated knockdown of PLK1 decreases the level of PLK1 protein in both Daoy and ONS-76 cells. [file 1471-2407-12-80-S1.JPEG]

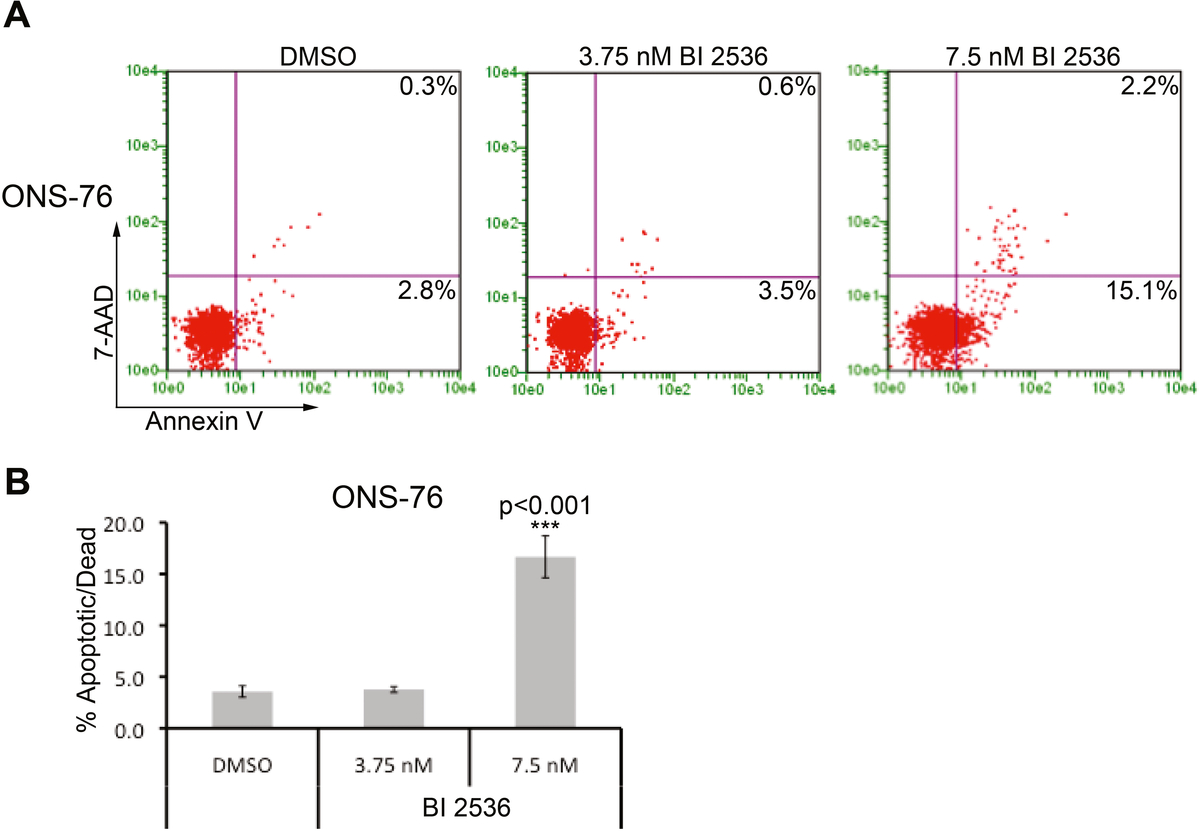

Supplement: Additional file 2 — Figure S2. Inhibition of PLK1 by the small molecule inhibitor BI 2536 increases apoptosis in ONS-76 cells. (A) Representative plots for ONS-76 cells stained with Guava Nexin reagent following 24 hours of treatment with BI 2536 or an equivalent amount of the drug's vehicle DMSO. Percentages of cells in the bottom left quadrant are live while cells to the right of the vertical line are apoptotic or dead. (B) The bar graph quantifies the average percentage of apoptotic/dead cells. Error bars represent SEM. [file 1471-2407-12-80-S2.JPEG]

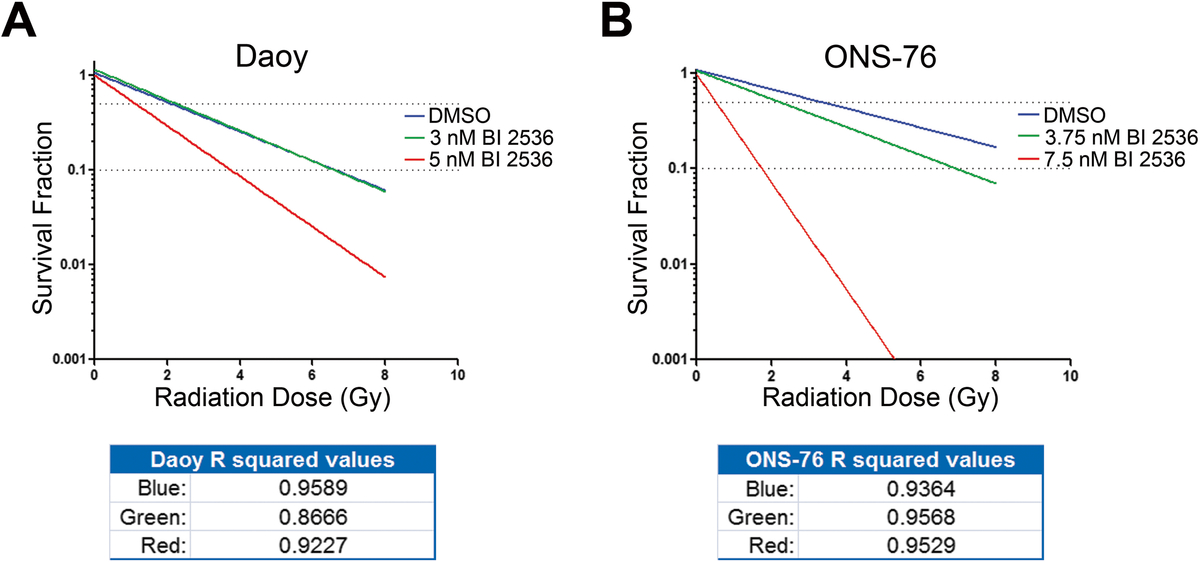

Supplement: Additional file 3 — Figure S3. Nonlinear regression lines for medulloblastoma cells treated with BI 2536 followed by radiation. (A) Daoy cells: the blue line is for DMSO treated cells, the green line for cells treated with 3 nM BI 2536, and the red line for cells treated with 5 nM BI 2536. (B) ONS-76 cells: the blue line is for DMSO treated cells, the green line is for cells treated with 3.75 nM BI 2536, and the red line is for cells treated with 7.5 nM BI 2536. The R2 values for each line are listed below their respective graphs. [file 1471-2407-12-80-S3.JPEG]

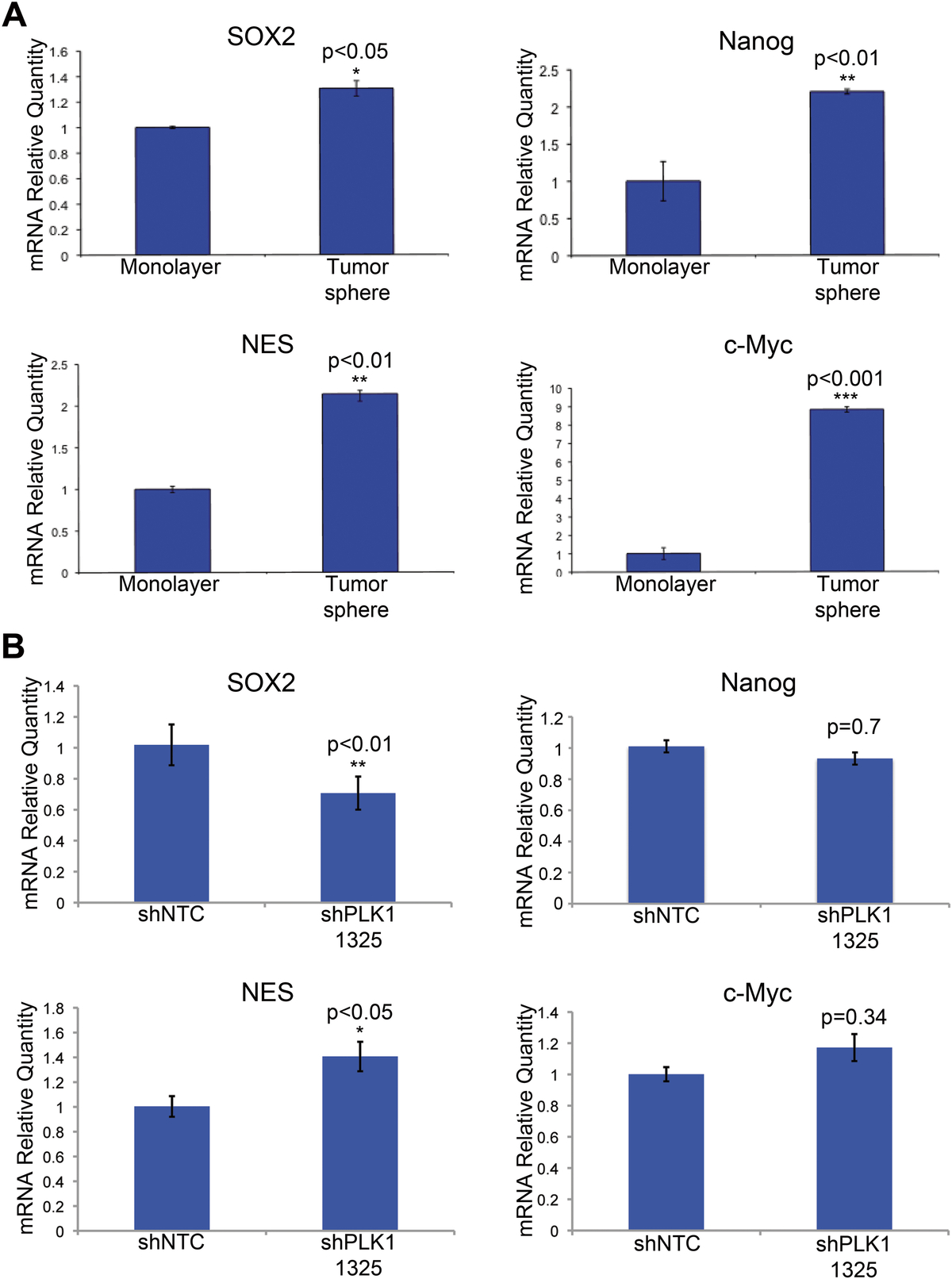

Supplement: Additional file 4 — Figure S4. Quantitative real-time PCR measure of the mRNA expression of stem/progenitor markers in Daoy cells. (A) Daoy cells were cultured under standard conditions or serum-free conditions. Stem/progenitor cell markers increase when cells are grown under serum-free tumor sphere conditions compared to standard serum containing adherent monolayer conditions. (B) Knockdown of PLK1 mRNA with shPLK1 1325 decreases SOX2 mRNA in Daoy cells grown as tumor spheres. There was no significant change in Nanog, NES, or c-Myc mRNA. [file 1471-2407-12-80-S4.JPEG]

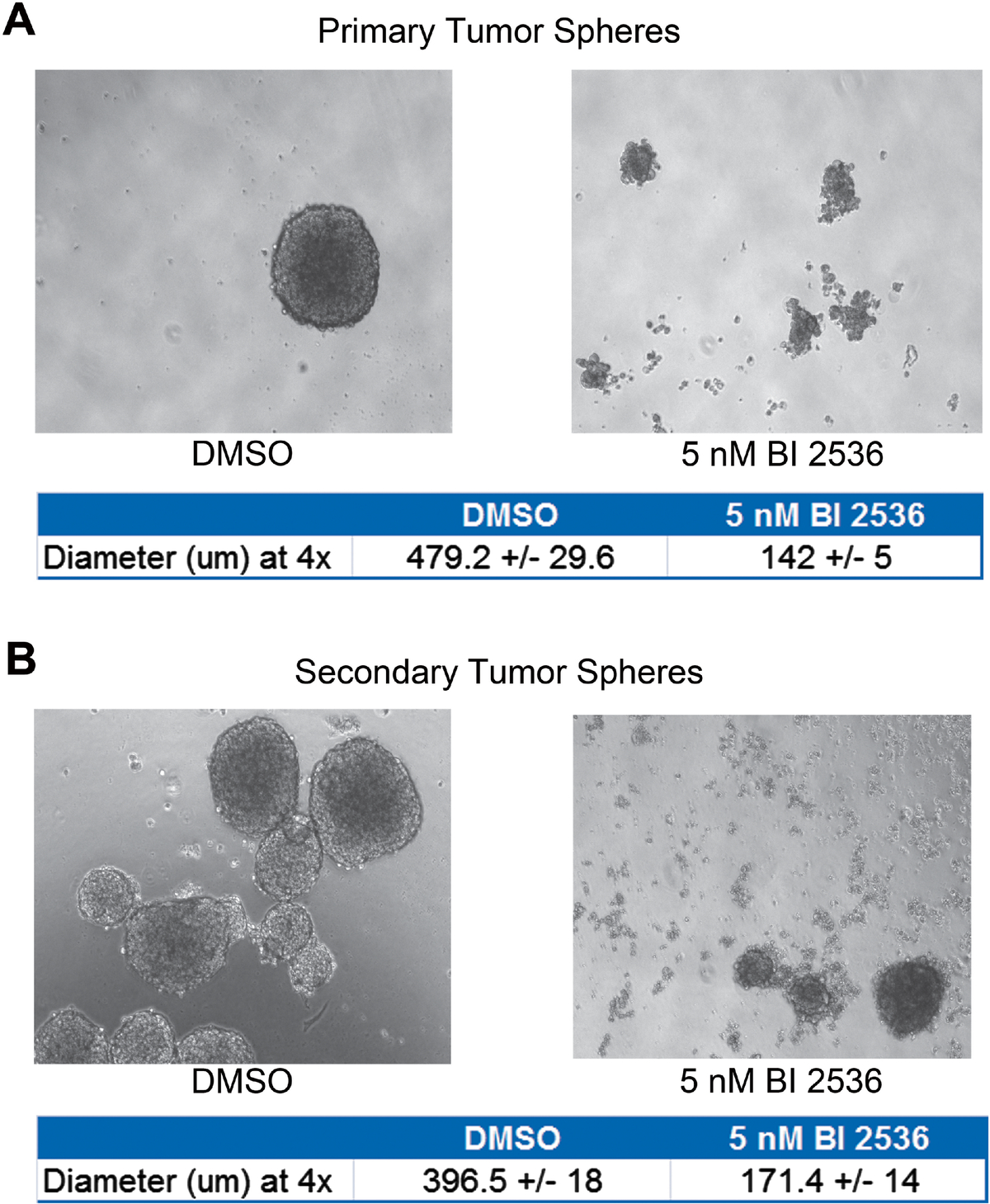

Supplement: Additional file 5 — Figure S5. BI 2536 decreases Daoy tumor sphere diameter. Daoy cells treated for 48 hours with 5 nM BI 2536 show decreased tumor sphere diameter in both (A) primary tumor spheres and (B) secondary tumor spheres. The diameter of the tumor spheres was measured using QCapture Pro software from saved images. Magnification 4×. [file 1471-2407-12-80-S5.JPEG]

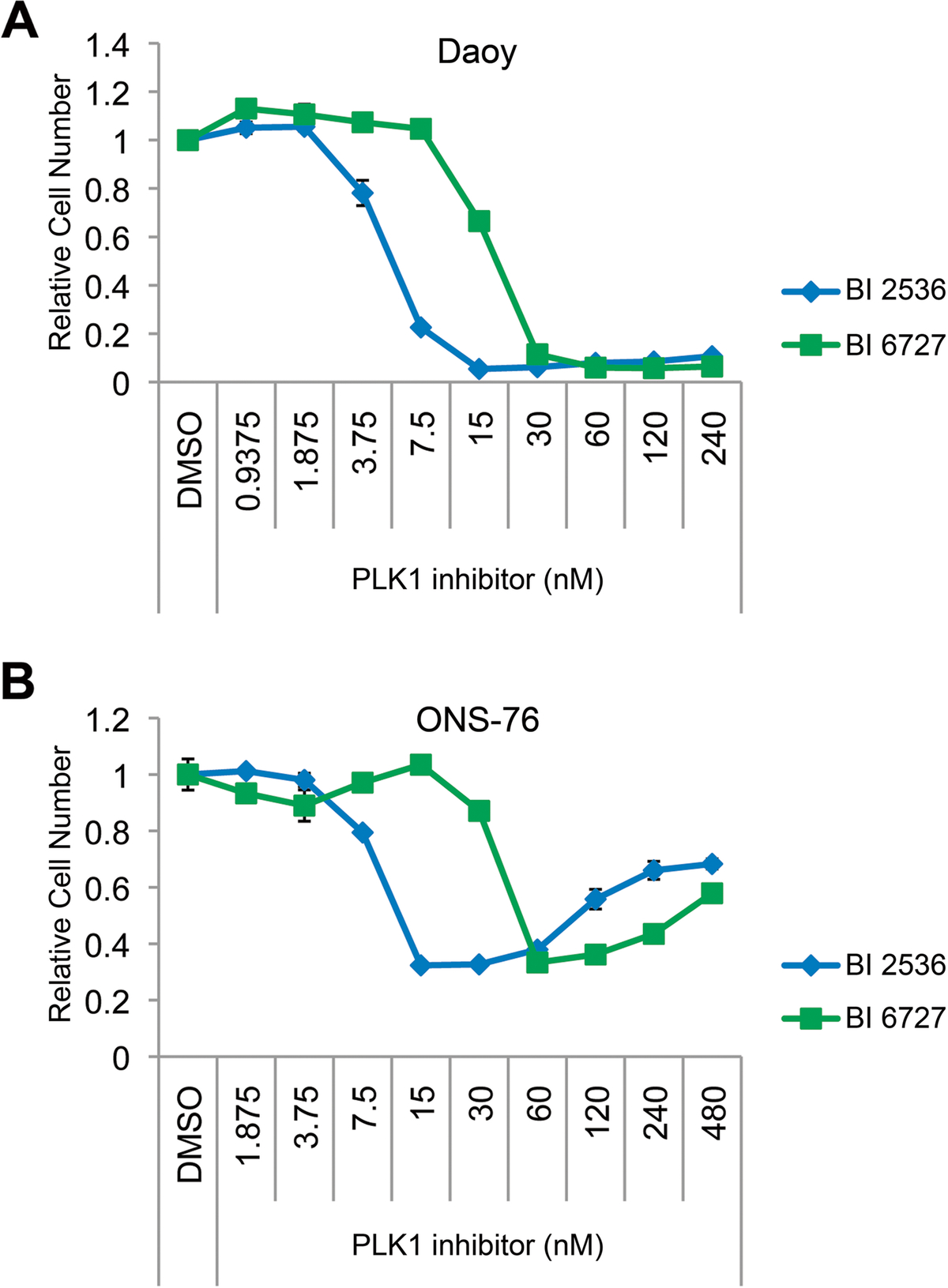

Supplement: Additional file 6 — Figure S6. Second generation PLK1 inhibitor BI 6727 induces growth arrest of medulloblastoma similar to BI 2536. Decrease in the relative cell number of two medulloblastoma cell lines, (A) Daoy and (B) ONS-76, treated for 72 hours with either BI 2536 (blue line and symbols) or BI 6727 (green line and symbols) as measured by MTS assay. Error bars represent SEM. [file 1471-2407-12-80-S6.JPEG]
